# Somatic Symptoms among Children and Adolescents in Poland: A Confirmatory Factor Analytic Study of the Children Somatization Inventory

**DOI:** 10.3389/fpubh.2013.00072

**Published:** 2013-12-24

**Authors:** Cecilia A. Essau, Beatriz Olaya, Anna Bokszczanin, Catherine Gilvarry, Diane Bray

**Affiliations:** ^1^Department of Psychology, University of Roehampton, London, UK; ^2^CIBER en Salud Mental (CIBERSAM), Barcelona, Spain; ^3^Parc Sanitari Sant Joan de Déu, Sant Boi de Llobregat, Barcelona, Spain; ^4^Opole University, Opole, Poland

**Keywords:** Children’s Somatization Inventory, anxiety disorders, somatic symptoms, Poland, children and adolescents

## Abstract

The aim of the present study was to examine the factor structure and psychometric properties of the short version of the Children’s Somatization Inventory (CSI-24) in Poland. The CSI-24 is a self-report questionnaire designed to assess somatic symptoms in children and adolescents. A total of 733 children and adolescents, aged 12–17 years, participated in this research. The participants for this study were recruited from urban and suburban schools of Opole province in South Western Poland. In addition to the CSI-24, all participants completed the Spence Children’s Anxiety Scale (SCAS) and the Strength and Difficulties Questionnaire (SDQ). The correlated four-factor model that included four-correlated dimensions (pain/weakness, gastrointestinal problems, cardiovascular symptoms, and pseudoneurological problems) showed a better fit compared to the single-factor model. The Cronbach’s Alpha for the CSI-24 was 0.91. Somatic symptoms correlated significantly highly with the SCAS total scores and the SDQ emotional subscale, suggesting good construct validity. Somatic symptoms had low correlation with the SDQ behavioral problems symptoms, suggesting adequate discriminant validity. The CSI-24 reliably measured somatic symptoms in children and adolescents in Poland.

## Introduction

Somatic symptoms such as stomach aches and headaches occur frequently in children and adolescents in the general population (1, 2). Somatic symptoms are more prevalent in girls than in boys, and they tend to increase with age (3). Youths with somatic symptoms tend to experience impairment in academic and social functioning including a high level of absenteeism, poor academic achievement, and psychosocial difficulties (4). Furthermore, somatic symptoms are linked to an increase in health care services utilization, thus, they tend to exert a heavy financial burden on both the families and the health care system (4).

Somatic symptoms co-occur frequently with anxiety and depression (5) and that they tend to be stable over time (6). Chronic somatic symptoms in childhood significantly predicts psychopathology in adulthood (7).These observations highlight the importance of understanding and screening somatic symptoms in children and adolescents.

The Children’s Somatization Inventory [CSI; (8)] is a self-report questionnaire for assessing somatic symptoms in children and adolescents. Its original version consists of 35 items (referred to as CSI-35) from which items were taken from the symptoms of somatization disorder (9), the somatization factor of the Hopkins Symptom Checklist (10), and common symptoms of functional gastrointestinal (GI) disorder (i.e., constipation). Children and adolescents report the extent to which they experience each symptom in the past 2 weeks on a 5-point scale ranging from “not at all” (0) to “a whole lot” (4). Somatization can be defined as a psychological distress that is manifested in the form of somatic symptoms.

Studies that examined the factor structure of the CSI-35 have reported considerable inconsistency regarding the factor structure of this instrument. For example, studies conducted in the US (11) and in the Ukraine (12) reported a four-factor structure on the CSI: pseudoneurological, cardiovascular, GI, and pain/weakness symptoms. However, in a study in the Netherlands (13) and the UK (3), only three factors were found: pain/weakness, GI, and pseudoneurological. In all of these studies, some of the CSI items are rarely endorsed and the item-total correlations were low. This is not surprising because some of the items were taken from the symptoms criteria for somatization disorders in adults which may not be applicable to children (14).

In a recent study by Walker and colleagues (14) that examined the dimensionality of the CSI-35, 11 statistically weak items (lump in throat, deafness, double vision, blindness, fainting/passing out, memory loss/amnesia, seizures, convulsion, trouble walking, paralysis/muscle weakness, difficulty urinating, pain-urinating) were deleted, leaving 24 items (referred to as CSI-24). The authors conducted a principal component analysis and found a single large component which explained almost 30% of the total variance and containing symptoms of various organs; a second factor containing symptoms related to GI symptoms was also found, but it was regarded as weak. The authors also conducted a confirmatory factor analysis (CFA) and found that the CSI-24 did not fit a single-factor model. Because of its better psychometric properties, shorter time completion and containing more appropriate items for children and adolescents, the authors suggested that the CSI-24 is preferable to the CSI-35.

In view of the above findings, it remains unclear whether a general somatization factor exists or whether somatic symptoms would be better represented by multidimensionality. Therefore, the main aim of this study was to test the factor structure of the Polish translation of the CSI-24: one-factor model (14), three-factor model (3, 13), four-factor model (11, 12), and two models including a general factor and specific group factors (i.e., bifactor models). A bifactor model would include a general factor that accounts for relationships between items, but also specific factors that account for the unique variance among the items above and beyond the general factor (15). Other aims of this study were to examine the relationship of somatic symptoms with other psychopathology, and to examine gender and age patterns of somatic symptoms.

## Materials and Methods

### Participants

The participants for this study were recruited from five urban and suburban schools of the Opole province in South Western Poland. A total of 733 adolescents participated in the study and of these 51.1% were girls. They ranged in age from 12 to 17 years (mean = 15.4, SD = 1.59). About 89.5% of the participants indicated Christianity as their religion.

Consent from the parent was obtained before the youths could participate in the study. Each participating school was sent a letter that described the aim of the present study and the procedure involved. The youths were informed about the aim of the study as well as about the voluntary nature and anonymity of their participation in this study. The questionnaires were completed by the participants in groups in the designated classrooms. A research assistant was available to provide answers if necessary and to ensure independent responding. Approval to conduct this study was obtained from the Psychology Ethic Committee at the University of Roehampton.

### Measures

In addition to the CSI-24, the participants also completed a brief questionnaire to obtain demographic characteristics, the Strengths and Difficulties Questionnaire (SDQ) (16), and the Spence Children’s Anxiety Scale (SCAS) (17). The English version of these questionnaires was adapted and translated to Polish according to guidelines that are widely accepted for the successful translation of instruments in cross-cultural research (18).

*The Strengths and Difficulties Questionnaire* (16) was used to assess general difficulties and positive attributes. Its 25 items are divided into 5 scales, which generate scores for conduct problems, hyperactivity-inattention, emotional symptoms, peer problems, and pro-social behavior; the “emotional symptoms” subscale assesses anxiety and depressive symptoms. Each of the items is rated on a 3-point scale, ranging from “not true” (0) to “certainly true” (2). In the present study, the Cronbach’s Alpha for the total SDQ scores was 0.79.

*The Spence Children’s Anxiety Scale* (17) is a 38-item measure of anxiety symptoms in children and adolescents. The items reflect symptoms of the main DSM-IV anxiety disorders, including separation anxiety, social phobia, obsessive-compulsive disorder (OCD), panic/agoraphobia, physical injury fears, and generalized anxiety disorder (GAD). Each item is rated on a 4-point scale in terms of its frequency from “never” (0) to “always” (3). In the present study, the Cronbach’s Alpha for the SCAS was 0.93.

### Statistical analysis

The Statistical Package for Social Science (SPSS) version 19.0 and the Structural Equation Modeling package EQS version 6.1 were used to perform the statistical analyses. The CSI-24 data were 99.2% complete, with 96% of cases having no missing items. With less than the 5% of cases having any missing data, any reasonable method of dealing with missing values could be used (19). Missing values were replaced using expectation maximization (EM) algorithm (20) to fulfill the 4% of missing items based on non-missing responses (21). This imputation method is thought to be better than the mean imputation since it preserves the variance. The average item mean and standard deviation were the same both before and after imputation (*M* = 0.62, SD = 0.93).

Corrected item-total correlations were calculated to examine how each item contributed to the overall scale. The Cronbach’s alpha was calculated to determine the internal consistency of the CSI-24.

**Figure 1:**
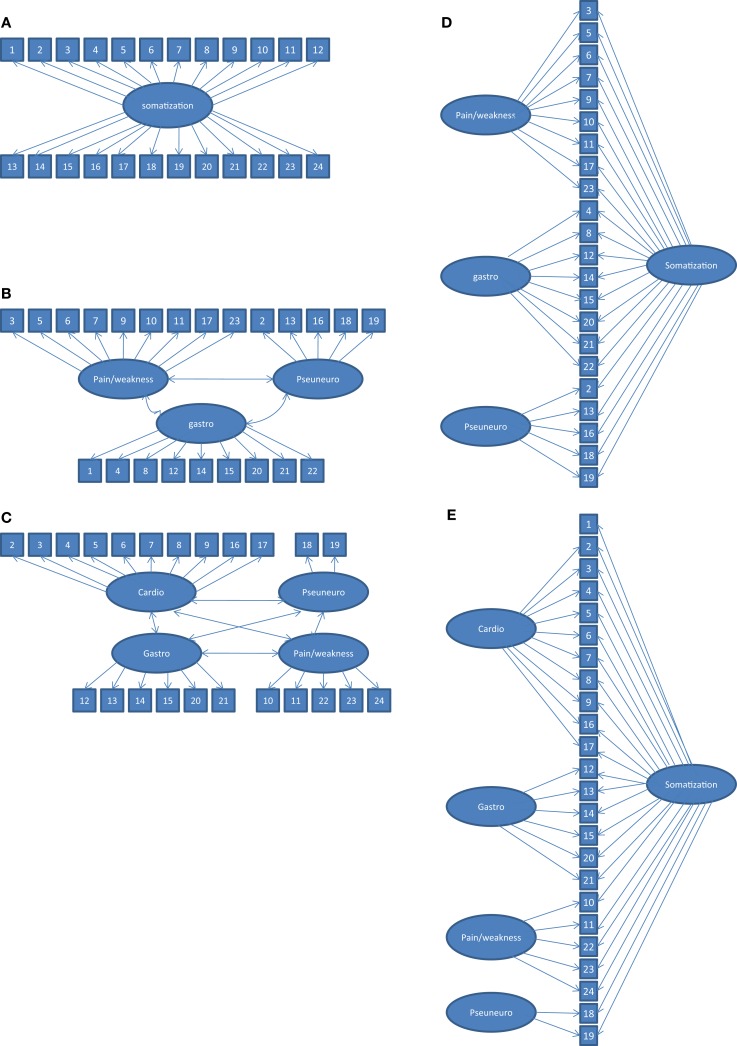
**Five factor models tested with CFA**. **(A)** unidimensional model (14). **(B)** three-correlated factor model (3, 13). Item 16 (“heart beating”) was considered as part of the pseudoneurological symptoms (3). **(C)** four- correlated factor model (11, 12). Item 1 (“headaches”) was not included as was proposed by the authors. **(D)** bifactor model with one general factor (somatization) and three specific factors (pain/weakness, gastrointestinal problems, and pseudoneurological problems). **(E)** bifactor model with one general factor (somatization) and four factors (Pain/weakness, cardiovascular problems, gastrointestinal problems, and pseudoneurological problems). This model included item 1 (“headaches”) as being part of the general factor.

Confirmatory factor analysis was performed to compare the one, three, and four-factor structure of the CSI (Figures 1A–C). The last two factors were considered to be factor-correlated. The Lagrange Multiplier tests (LM test) were also conducted to determine unspecified parameters in the model. Statistically significant LM Chi-square values would argue for the presence of factor cross-loadings and error covariances (22). Two bifactor models were specified in which each item loaded onto a general somatization factor, and items also loaded onto one of the three or the four domain-specific factors (Figures 1D,E).

Since the observed variables included in the model were ordinal and presented a certain level of skewness, a maximum likelihood robust method based on polychoric correlations was used (23). Satorra–Bentler scaled Chi-square, the Normed Fit Index (NFI), the Non-Normed Fit Index (NNFI), the Comparative Fit Index (CFI), the Root Mean Square Error of Approximation (RMSEA), the Standardized Root Mean Square Residual (SRMR) were used as goodness-of-fit indices. Values of NFI, NNFI, and CFI higher than 0.90 indicate adequate fit (24). RMSEA of 0.05 or lower is indicative of an adequate fit (25); a favorable value of the SRMR is less than 0.10 (26). Robust versions of all fit statistics were computed except for the SRMR, which has no robust counterpart but summarizes the fit in a way the other (robust) indices do not. The Akaike’s Information Criterion (AIC), the consistent AIC (CAIC), and the Expected Cross-Validation Index (ECVI) were used as parsimony indices: smaller model AIC, CAIC, and ECVI than the comparison model indicate better fit.

Non-parametric correlation tests (Spearman rank) were computed between the CSI and the SCAS and the SDQ to assess the convergent and divergent validity of the CSI. Gender and age effects were examined with Mann–Whitney and Kruskal–Wallis tests.

## Results

Descriptive analyses of the CSI-24 items (Table 1) showed the lowest mean for items “difficulty swallowing,” “losing voice,” and “vomiting.” The highest mean was obtained for “headaches,” followed by “low energy,” “pain in stomach or abdomen.” There was a marked skewness or kurtosis for the following items: “constipation,” “loose BM’s or diarrhea,” “difficulty swallowing,” “losing voice,” “vomiting,” and “food making sick.” All items obtained a corrected item-total correlation greater than the rule of thumb minimum value of 0.20 (27).

**Table 1:** **Characteristics of all the CSI-24 items**.

CSI-24	Symptoms endorsed at any level of severity (%)	Symptoms endorsed as 3 (a lot) or 4 (a whole lot) (%)	Mean	SD	Skewness	Kurtosis	*r* tot
1. Headaches	70.4	12.1	1.32	1.09	0.36	−0.58	0.50
2. Faintless or dizziness	41.1	6.2	0.70	1.00	1.38	1.24	0.62
3. Pain heart or chest	38.2	7.3	0.68	1.03	1.53	1.63	0.55
4. Low energy	63.1	11.6	1.13	1.13	0.85	0.02	0.54
5. Pains lower back	43.8	13.2	0.89	1.19	1.12	0.07	0.48
6. Sore muscles	57.7	9.1	1.01	1.09	0.93	0.21	0.50
7. Trouble breath	26.0	4.7	0.46	0.90	2.24	4.72	0.61
8. Hot or cold spells	38.2	9.1	0.72	1.10	1.52	1.41	0.60
9. Numbness or tingling	48.6	10.4	0.87	1.10	1.14	0.40	0.59
10. Weakness	34.4	4.7	0.54	0.89	1.80	2.92	0.62
11. Heavy feelings arms or legs	27.0	2.3	0.39	0.76	2.36	6.23	0.56
12. Nausea or upset stomach	40.6	7.6	0.71	1.05	1.49	1.50	0.64
13. Constipation	17.2	2.6	0.27	0.68	3.10	10.35	0.48
14. Loose BM’s or diarrhea	16.9	2.6	0.27	0.73	3.39	12.49	0.43
15. Pain stomach or abdomen	62.3	10.7	1.06	1.06	0.82	−0.04	0.53
16. Heart beating fast	27.3	5.4	0.48	0.92	2.20	4.44	0.57
17. Difficulty swallowing	12.8	2.3	0.22	0.67	3.57	13.57	0.53
18. Losing voice	13.9	1.6	0.22	0.62	3.58	14.57	0.41
19. Blurred vision	23.9	3.3	0.39	0.81	2.45	6.19	0.44
20. Vomiting	12.8	1.6	0.22	0.65	3.37	12.11	0.40
21. Floated or gassy	36.3	8.0	0.67	1.07	1.69	2.10	0.35
22. Food making sick	21.1	2.5	0.33	0.75	2.77	8.36	0.61
23. Pains knees, elbows, or joints	41.6	9.1	0.77	1.09	1.34	0.91	0.46
24. Pain arms or legs	38.4	6.2	0.64	1.00	1.72	2.51	0.58

**r* tot, Corrected item-total correlation; the standard error of skewness was 0.09 and 0.18 for kurtosis*.

### Confirmatory factor analyses

Lagrange multiplier test in the one-factor model showed that the highest LM Test incremental multivariate Chi-square values represent error covariances between items 13 (“constipation”) and 14 (“Loose BM’s or diarrhea”), items 14 (“diarrhea”) and 22 (“Food intolerance”), items 13 (“constipation”) and 21 (“floated or gassy”), items 14 (“diarrhea”) and 21 (“floated or gassy”), and between item 23 (“pain joints”) and item 24 (“pain in arms and legs”). To understand these results, it is important to look at the content of these items. It seems that there might be an overlap between them (i.e., pain in joints and pain in arms or legs), and in other cases, items refer to symptoms that occur generally at the same time (i.e., constipation and being gassy). Therefore, the one-factor model was re-specified including these parameters.

For the three-factor model, the LM-test showed the following misspecified parameters (error covariances): items 13 (“constipation”) and 14 (“diarrhea”), items 14 (“diarrhea”) and 21 (“floated or gassy”), items 13 (“constipation”) and 21 (“floated or gassy”), and items 23 (“pain joints”) and 24 (“pain in arms and legs”). The LM-test in the four-factor model included as misspecified parameters the following error covariances: 23 (“Pain in knees, elbows, or joints”) and 24 (“Pain in arms and legs”), items 13 (“constipation”) and 14 (“Loose BM’s or diarrhea”), items 14 (“diarrhea”) and 21 (“floated or gassy”), and items 13 (“constipation”) and 21 (“floated or gassy”). Moreover, the LM-test showed as misspecified a cross-loading from factor 4 (pseudoneurological) to item 17 (“difficulty swallowing”), and from factor 2 (GI problems) to item 22 (“Food intolerance”). After reviewing the content of each item, it was considered that these error covariances and cross-loadings should be kept in both the three- and four-factor models including these new parameters. The goodness-of-fit indices of the new re-specified models were better than the models without these items, and the different values of the Satorra–Bentler Chi-Squared [calculated with the computation process from (28)] were also significant (*p* < 0.001).

Table 2 includes the goodness-of-fit indices with these new parameters in the three models. Overall, the goodness-of-fit indices were good in all models under test. However, the model which yielded better fit indices as well as better parsimonious indices was the four-factor model. Bifactor model including the three domain-specific factors (i.e., GI, pseudoneurological, and pain/weakness) showed better indices than the three-factor model, whereas goodness-of-fit of the bifactor model with the four domain-specific factors was in general poorer than the four-correlated factors.

**Table 2:** **Goodness-of-fit indices for the one-, three-, four-factors, and bifactor models of the CSI-24**.

Fit indices	1 Factor	3 Factor	4 Factor	Bifactor model (3 subfactors)	Bifactor model (4 subfactors)
Satorra–Bentler Chi-squared (df)	811.08 (247)	759.02	504.52 (218)	568.86 (221)	662.46 (229)
NFI	0.965	0.968	0.977	0.976	0.972
NNFI	0.973	0.975	0.985	0.981	0.977
CFI	0.976	0.978	0.987	0.985	0.981
RMSEA (95% CI)	0.056 (0.052–0.060)	0.054 (0.049–0.058)	0.042 (0.038–0.047)	0.046 (0.042–0.051)	0.051 (0.046–0.055)
SRMR	0.060	0.059	0.052	0.50	0.055
Model AIC	317.08	269.02	68.52	126.87	204.46
Model CAIC	−1065.42	−1102.28	−1151.661	−1110.10	−1077.28
ECVI	3.22	3.09	2.18	2.52	2.78

*df, degrees of freedom; CI, confidential interval*.

Factors loadings in the one-factor model ranged from 0.45 (item 21) to 0.78 (item 17) (Table 3). In the three-factor and four-factor models, items showed high loadings on their respective hypothesized factors. In the four-factor model, however, items 17 (“difficulty swallowing”) and 22 (“food intolerance”) had low values in their cross-factor loading (0.17 and 0.15, respectively), which were marginally significant. Factor loadings to the general factor in the bifactor models tend to go down slightly compared with the loadings in the one-factor solution. This might occur because the factor in the unidimensional model is likely a conglomeration of the common variance present through all items due to shared content (29). In the bifactor model with three specific group factors, 15 out of 24 items had significant factor loadings on the general factor as well as on the specific factors. Particular symptoms did not share symptom specific variances and only loaded significantly on the general factor (e.g., “trouble breathing” and “heart beating fast”). Similarly, in the bifactor model with four specific group factors, 14 out of 24 items showed significant factor loadings on both the general factor and the specific group factor. Again, some items did not significantly load onto the specific factor (e.g., “sore muscles” or “difficulty swallowing”). Inter-factor correlations were very high, both in the three-factor and the four-factor model.

**Table 3:** **Factor-loadings and inter-factor correlations for the CSI-24**.

	One-F	Three-F model			Four-F model				Bifactor Model (3 subfactors)				Bifactor model (4 subfactors)				
	F1	F1	F2	F3	F1	F2	F3	F4	*g*	F1	F2	F3	*g*	F1	F2	F3	F4
CSI1	0.56*		0.57*						0.57*		−0.11		0.55*				
CSI2	0.71*			0.70*	0.72*				0.74*			−0.19*	0.68*	0.29*			
CSI3	0.66*	0.67*			0.69*				0.69*	−0.03			0.61*	0.51*			
CSI4	0.62*		0.60*		0.63*				0.60*		−0.07		0.62*	0.04			
CSI5	0.56*	0.57*			0.57*				0.50*	0.26*			0.56*	0.04			
CSI6	0.54*	0.55*			0.55*				0.44*	0.40			0.54*	0.02			
CSI7	0.75*	0.75*			0.78*				0.75*	0.02			0.72*	0.31*			
CSI8	0.71*		0.70*		0.74*				0.72*		0.003		0.69*	0.26*			
CSI9	0.67*	0.68*			0.68*				0.63*	0.18*			0.67*	0.08			
CSI10	0.74*	0.74*					0.76		0.70*	0.16*			0.74*			0.04	
CSI11	0.69*	0.70*					0.74		0.59*	0.34*			0.69*			0.17*	
CSI12	0.75*		0.82*			0.82*			0.79*		0.28*		0.76*		0.05		
CSI13	0.62*			0.62*		0.60*			0.61*			0.08	0.62*		0.40*		
CSI14	0.57*		0.61*			0.64*			0.60*		0.40*		0.57*		0.75*		
CSI15	0.61*		0.65*			0.65*			0.63*		0.19*		0.61*		0.03		
CSI16	0.72*			0.71*	0.73*				0.73*			0.005	0.69*	0.25*			
CSI17	0.78*	0.79*			0.17	0.71*			0.75*	0.08			0.78*	0.01			
CSI18	0.60*			0.61*				0.71*	0.58*			0.45*	0.60*				0.57*
CSI19	0.60*			0.60*				0.67*	0.60*			0.29*	0.59*				0.23*
CSI20	0.60*		0.66*			0.70*			0.66*		0.59*		0.62*		0.25*		
CSI21	0.45*		0.45*			0.47*			0.45*		0.24*		0.45*		0.45*		
CSI22	0.76*		0.80*			0.69*	0.15		0.79*		0.31*		0.78*			−0.01	
CSI23	0.51*	0.53*					0.54*		0.39*	0.47*			0.51*			0.47*	
CSI24	0.63*	0.65*					0.68*		0.49*	0.55*			0.62*			0.93*	
**INTER-FACTOR CORRELATIONS**																	
F1		1.00	0.90	1.00	1.00	0.83	0.91	0.79									
F2			1.00	0.94		1.00	0.80	0.78									
F3				1.00			1.00	0.83									
F4								1.00									

*g*, general factor; *Test statistics significant at the 5% level.

### Internal consistency and validity

The Cronbach’s alpha of the CSI-24 was 0.91. Internal consistency of the four-factor model was good in three of its subscales. Cronbach’s alpha coefficient of the GI factor was 0.72, for the cardiovascular problems was 0.84, and for the pain/weakness was 0.76. The internal consistency of the pseudoneurological factor was poor, with a Cronbach’s alpha of 0.48.

The CSI-24 total scores correlated highly with the SCAS total scores and all its subscales, as well as with the SDQ total scores and all its subscales except for the pro-social behavior subscale (Table 4). Correlations between the CSI-24 and the SDQ conduct problems and ADHD subscales were high (both equal to 0.40). In order to determine whether correlations between the CSI-24 and the SCAS and the SDQ emotional problems were significantly stronger (convergent validity) than correlations between the CSI-24 and the SDQ conduct problems and ADHD subscales (divergent validity), we used the Steiger’s *Z* test (30), following the recommendation of Meng et al. (31) for correlated correlations. Results showed that the relationship between the CSI-24 and the SCAS OCD, panic, GAD, SCAS total score, and SDQ emotional problems were significantly greater than the correlations between the CSI-24 and the SDQ conduct problems and the correlations between the CSI-24 and the SDQ hyperactivity (*z* > 1.96, *p* < 0.05). However, results showed that correlations between the CSI-24 and the SAD, Social phobia, and fears subscales were not significantly different from correlations between the CSI-24 and both the SDQ conduct problems and hyperactivity subscales (*z* < 1.96, *p* > 0.5).

**Table 4:** **Correlations between CSI-24 and other psychopathological measures**.

	CSI-24 total score
**SPENCE CHILDREN’S ANXIETY SCALE**	
Separation anxiety	0.39**
Social phobia	0.39**
Obsessive compulsive	0.49**
Panic disorder	0.59**
Physical injuries fears	0.36**
Generalized anxiety	0.48**
Total scores	0.56**
**STRENGTH AND DIFFICULTIES QUESTIONNAIRE**	
Emotional symptoms	0.59**
Conduct problems	0.40**
Hyperactivity-inattention	0.40**
Peer problems	0.24**
Pro-social behavior	0.03
Total scores	0.59**

**Significant at the 0.01 level (two-tailed).

### Gender and age effects

The mean of the CSI-24 total score on the whole sample was 14.95 (SD = 12.99). We also calculated the means and standard errors of the four factors: GI factor (mean = 3.21, SD = 3.47); cardiovascular (mean = 7.14, SD = 6.60), pain/weakness (mean = 2.67, SD = 3.24), and pseudoneurological factor (mean = 0.60, SD = 1.16). Girls had significantly higher mean scores than boys on the CSI-24 total score, GI, cardiovascular, and pseudoneurological symptoms (Table 5). Comparisons between three groups (12–13, 14–15, 16–17 years) in terms of the mean scores on the CSI-24 and the four subscales yielded no significant differences.

**Table 5:** **Means and standard deviations of the CSI-24 total score by age and gender groups**.

	CSI-24 total		Gastro		Cardio		Pain/weak		Pseudoneuro	
	**Mean (SD)**	***U* Mann– Whitney (*p*)**	**Mean (SD)**	***U* Mann– Whitney (*p*)**	**Mean (SD)**	***U* Mann– Whitney (*p*)**	**Mean (SD)**	***U* Mann– Whitney (*p*)**	**Mean (SD)**	***U* Mann– Whitney (*p*)**

Girls	16.52 (13.71)	51126 (*p* < 0.001)	3.52 (3.32)	54060 (*p* < 0.001)	7.94 (6.49)	52200 (*p* < 0.001)	2.77 (3.15)	59016 (*p* = 0.06)	0.66 (1.16)	58043 (*p* < 0.01)
Boys	14.68 (12. 60)		2.96 (3.65)		6.43 (6.73)		2.17 (3.36)		0.54 (1.18)	

		**Kruskal–Wallis (df; *p*)**		**Kruskal–Wallis (df; *p*)**		**Kruskal–Wallis (df; *p*)**		**Kruskal–Wallis (df; *p*)**		**Kruskal–Wallis (df; *p*)**

12–13 years	13.96 (11.43)	0.59 (2; 0.74)	3.06 (2.99)	1.15 (2; 0.56)	6.53 (5.91)	1.16 (2; 0.56)	2.67 (3.01)	1.72 (2; 0.42)	0.49 (0.79)	0.27 (2; 0.87)
14–15 years	14.68 (12.60)		3.11 (3.51)		6.93 (6.42)		2.74 (3.08)		0.56 (1.08)	
16–17 years	15.50 (13.79)		3.33 (3.60)		7.47 (6.94)		2.66 (3.46)		0.67 (1.31)	
**GIRLS**										
12–13 years	15.29 (11.77)	1.58 (2; 0.45)	3.42 (3.30)	0.89 (2; 0.64)	7.15 (6.14)	1.80 (2; 0.41)	2.70 (2.93)	0.06 (2; 0.97)	0.56 (0.79)	0.15 (2; 0.93)
14–15 years	15.41 (12.05)		3.30 (3.33)		7.27 (6.04)		2.61 (2.92)		0.65 (1.15)	
16–17 years	17.17 (12.60)		3.59 (3.28)		8.32 (6.75)		2.85 (3.35)		0.71 (1.25)	
**BOYS**										
12–13 years	12.80 (11.12)	1.05 (2; 0.59)	2.78 (2.70)	0.34 (2; 0.84)	6.05 (5.79)	0.46 (2; 0.80)	2.54 (2.89)	4.88 (2; 0.09)	0.40 (0.75)	0.53 (2; 0.76)
14–15 years	14.01 (13.13)		2.95 (3.69)		6.61 (6.78)		2.84 (3.24)		0.47 (1.02)	
16–17 years	13.56 (14.93)		3.04 (3.94)		6.47 (7.08)		2.43 (3.60)		0.64 (1.38)	

Due to the violation of the normality assumption, Age × Gender interaction was not examined. However, we examined the mean scores of the CSI-24 among boys and girls separately within the three age groups. There were no significant differences in the different CSI scores between age groups according to the participants’ gender (Table 5).

## Discussion

The main purpose of this article was to examine the factor structure, reliability, and the validity of the Polish version of the CIS-24. Another aim was to examine the effects of gender and age on the frequency of somatic symptoms. Our main findings are summarized as follows: first, the better fit of the correlated four-factor model compared to the single-factor model supports the multicomponential nature of the CSI-24 with four-correlated dimensions: pain/weakness, GI problems, cardiovascular symptoms, and pseudoneurological problems. This factor structure corresponded to the factor structure reported by Garber et al. (11) and Litcher et al. (12) when the original CIS-35 was used with children and adolescents in the US and Ukraine. However, our findings were inconsistent with those by Walker et al. (14) in which one dominant general factor was found when a PCA was conducted. However, the bifactor models under test yielded good fit indices and the inspection of the factor loadings showed that many symptoms significantly loaded onto both the general factor and the specific factors. This would suggest that the variability of a CSI-24 symptom is determined by two sources of systematic variance, one from a general somatization factor and the other from a specific factor. Some authors have suggested that this could be explained in the light of the presence of an affective component associated to the symptom experience as the general factor, whereas the specific factors would correspond to the sensory part of the symptom experience (32). Another point of concern is the high inter-factor correlations between the three and the four factors, which could be suggestive of the presence of a second-order factor. In such a model, correlations between factors would be accounted for by the general somatization factor. The authors conducted a CFA with second-order factor models (with both the three and the four factors), although results did not convergent to admissible solution. If this model provides better fit to the data, the domain would consist of a single broad somatization construct that can be broken down into specific manifestations or facets. Thus, it is possible to calculate a total score of somatic symptoms by adding up all the items as well as to use different dimensions that might better reflect the complexity of the somatization construct in children and adolescents.

Second, the Cronbach’s alpha of the CSI-24 was 0.91. Studies conducted in the US and Turkey (14, 33) have similarly shown high internal consistency for the CSI-24, with Cronbach’s Alphas ranging from 0.90 to 0.91. Third, similar to previous studies (3), girls reported significantly higher number of somatic symptoms than boys. The reason for this gender difference is unclear although biological factors related to puberty (e.g., menstruation) have been considered as contributing to higher somatic symptoms in girls (34). The role of age in somatic symptoms is less clear. In the study by Vila et al. (3) in the UK, the 11- to 12-year-olds had significantly higher scores than the 13- to 14-year-olds. It was argued that the 11- to 12-year-olds were more stressed than the 13- to 14-year-olds as they needed to adapt to a new school.

Fourth, in agreement with several studies (3), the Polish CSI-24 has a good convergent validity. Specifically, the CSI-24 total score correlated significantly with the SCAS, and the SDQ, suggesting that a high level of somatic symptoms was associated with a high level of anxiety and emotional problems. The CSI-24 showed good construct validity as evidenced by the significant positive correlations with the anxiety symptoms as measured by the SCAS and on the emotional subscale of the SDQ. These findings are consistent with previous studies that showed significant correlation between somatic and two internalizing problems [i.e., anxiety, and emotional symptoms (35)]. In agreement with previous studies (3), somatic symptoms had low correlation with behavioral problems (which is an example of an externalizing problem), suggesting adequate discriminant validity. These findings seem to suggest that the CSI-24 can be used to distinguish internalizing from externalizing problems. Internalizing problems are inner-directed which cause distress to oneself, while as externalizing problems are outer-directed which cause distress to others (36).

There are several limitations to the present study, which need to be taken into consideration when interpreting our results. First, our participants were 12- to 17-year-olds who were recruited from school settings. The use of a community sample may have implications for the generalizability of our findings to other populations such as those in clinical setting or younger age groups. Second, the data were based on self-report; however children and adolescents have been reported to be better informants than parents because of the internalizing experiences of some somatic symptoms. Indeed studies have reported disagreement between children and their parents when reporting somatic symptoms (37), with mothers reporting more somatic symptoms than the children. Third, because the CSI-24 does not provide information on possible medical explanations for symptoms, it is unclear whether or not the symptoms reported on the CSI-24 are an expression of organic problems. These limitations notwithstanding, our findings support the usefulness of the CSI-24 as an efficient way of screening for somatic symptoms in children and adolescents in Poland.

Future studies should replicate the present study in other settings (e.g., clinical setting) and age groups.

## Conflict of Interest Statement

The authors declare that the research was conducted in the absence of any commercial or financial relationships that could be construed as a potential conflict of interest.
